# Optimal solution to the set cover problem with a vicinity constraint for estimating genotype tissue expression profiles

**DOI:** 10.1093/bioadv/vbaf163

**Published:** 2025-07-04

**Authors:** Jiahong Dong, Stephen Brown, Kevin Truong

**Affiliations:** Edward S. Rogers, Sr. Department of Electrical and Computer Engineering, University of Toronto, Toronto, ON M5S 3G4, Canada; Edward S. Rogers, Sr. Department of Electrical and Computer Engineering, University of Toronto, Toronto, ON M5S 3G4, Canada; Edward S. Rogers, Sr. Department of Electrical and Computer Engineering, University of Toronto, Toronto, ON M5S 3G4, Canada; Institute of Biomedical Engineering, University of Toronto, Toronto, ON M5S 3E3, Canada

## Abstract

**Motivation:**

Genes located in close genomic proximity tend to have more similar genotype tissue expression profiles. This suggests that expression profiles for the entire genome could be estimated using a smaller set of experimentally determined profiles from carefully selected reference genes, thereby reducing the need for extensive experimental measurements.

**Results:**

We address this challenge by mapping it as a set cover problem, aiming to identify an optimal number of gene sets that can cover the entire genome. However, traditional set cover algorithms are either slow in runtime or yield non-optimal results for large datasets. To overcome this limitation, we developed a dynamic programming algorithm that leverages the consecutive ordering of genes within vicinity sets. Our algorithm solves this vicinity set cover problem with tractable runtime while minimizing the average distance between reference genes and non-reference genes within the vicinity, thereby maximizing estimation accuracy. This algorithm can be used to reduce the number of required experiments in organisms lacking genotype tissue expression data or in new human datasets with expanded tissue sets. Lastly, our algorithm also has broader applications for set cover optimization problems in other fields.

**Availability and implementation:**

The source code along with all implementation details are available at: https://github.com/sensationTI/vicinity_set_cover.

## 1 Introduction

In computer science, the set cover problem is often used as a benchmark for hardness in complexity theory studies and its solutions have applications in network design (Agathos and Papapetrou 2013), logistics (Boschetti and Maniezzo 2015), and bioinformatics (Heger and Holm 2001, Stoney *et al.* 2018, Savage *et al.* 2019, Li *et al.* 2020). In the set cover problem, a universe of elements is contained within a collection of n sets whose union covers the entire universe of elements. The optimal solution is the smallest number of k sets to cover the entire universe of elements. Algorithms to solve the set cover problem are either optimal or approximate, where optimal solutions are NP-hard (Caprara *et al.* 2000, Krishnaswamy *et al.* 2017, Carniel and Mestria 2023). Optimal solutions, like the branch and bound algorithm, systematically explore the solution space using a tree structure, branching into smaller subproblems. The branch and bound algorithm can find an optimal solution but the worst-case time complexity is NP-hard (Dey *et al.* 2023). In contrast, the family of greedy algorithm always selects the sets that maximizes the number of uncovered elements at each iteration and has complexity of O($n^{2}$). Although it does not guarantee the optimal solution, it will find at most kln(n) sets, which is at most ln(n) times more than the optimal solution (Chvatal 1979). Although the set cover problem is NP-hard, there are variants that have bioinformatic applications such as when the elements are consecutively ordered to define a vicinity (hereafter, called vicinity set cover problem).

In recent studies, it was shown that genotype tissue expression profiles are more similar if they are located in a closer vicinity (Dong *et al.* 2024). The genotype tissue expression profile of a particular gene is created from the normalized expression level of that gene across a collection of tissues. For instance, the Genotype-Tissue Expression (GTEx) project has collected the expression of all genes in 54 non-diseased tissues sampled across nearly 1000 individuals, which can then be used to create the genotype tissue expression profile of every gene (GTEx Consortium 2013). When comparing expression profiles of genes located within the vicinity of 20 000 to 60 000 base pairs, there was a tendency toward more similar genotype tissue expression profiles, regardless of the gene orientation (i.e. sense or antisense) (Dong *et al.* 2024). This similarity in genotype tissue expression profiles also extended to non-coding genes (e.g. micro-RNA and small nuclear RNA). A rationale for this phenomena lies in the notion that if the chromatin is regulated to be open, then all genes in the vicinity will have potential for gene expression (Klemm *et al.* 2019). This apparent similarity in genotype tissue expression profile from vicinity leads to the possibility of estimating profile with fewer experiments.

GTEx acquires gene expression data by first doing whole genome sequencing to determine genotypes of particular individuals, followed by RNA sequencing (RNA-seq) to measure gene expression levels across various tissues. Although next generation sequencing technology has made RNA-seq more accessible (Hou *et al.* 2015, McCombie *et al.* 2019), large-scale projects like GTEx often require collaboration among consortia of researchers. It is helpful to have a method to estimate and survey genotype tissue expression profiles without conducting as many experiments as possible. Since neighboring genes tend to have more similar profiles when they are closer, we can estimate genotype tissue expression profiles of the entire genome with fewer experiments. In essence, this is solving the vicinity set cover problem, which we show hereafter can arrive at the optimal solution (given a threshold for the vicinity) in order complexity O($n^{2}$). In this case, the elements are the genes; the universe is the genome; the sets are genes in a vicinity where their expression profiles are deemed similar.

## 2 Methods

### 2.1 Pearson correlation coefficient calculation

We used the Pearson correlation coefficient (PCC) to quantify the similarity between the expression profiles of neighboring genes. This statistical measure quantifies the strength and direction of the linear relationship between two variables, ranging from -1 to +1. A PCC of -1 indicates a perfect negative correlation, while a value of +1 indicates a perfect positive correlation. When applied to gene expression profiles across 54 tissues, the expression values for two genes *x* and *y* in tissue i (measured in transcripts per million) are denoted as $x_{i}$ and $y_{i}$, respectively. Let $u_{x}$ and $u_{y}$ represent the average expression values of *x* and *y* across all tissues. The PCC between *x* and *y* is then calculated using the following:

PCC=∑i=154(xi-ux)(yi-uy)∑i=154(xi-ux)2∑i=154(yi-uy)2         (1)

### 2.2 Software and datasets

The human genotype tissue expression data used in this study were obtained from the GTEx Portal (https://gtexportal.org/home/datasets), specifically from the GTEx Analysis v8 release (dbGaP accession phs000424.v8.p2) (GTEx Consortium 2013). Launched by the NIH in 2010, the GTEx project provides a foundational resource for studying how genetic variation influences gene expression in non-diseased human tissues. Its most recent release, GTEx v8 (2020), includes over 17 000 RNA-seq samples across 54 tissue types from 838 postmortem donors, as well as single-nucleus RNA-seq (snRNA-seq), whole-genome sequencing (WGS). The data are typically provided in normalized forms such as transcript per million (TPM). However, many expression profiles are incomplete, missing, or display zero variance across tissues. As a result, such entries must be excluded when computing Pearson correlation coefficients to ensure valid and meaningful results. The mouse genotype tissue expression data were obtained from the Mouse Expression and Splicing Atlas (MESA), part of the Alternative Splicing Catalog of the Transcriptome (ASCOT) project developed at Johns Hopkins University (Ling *et al.* 2020). MESA is a transcriptomic reference for mouse tissues and cell types, analogous to GTEx in humans, which compiles bulk RNA-seq data from 732 purified mouse cell populations, covering 126 tissues. All data presented in this paper were processed and analyzed using the Python programming language (version 3.9.7).

### 2.3 Normalizing genotype tissue expression profile

Since the primary value of expression profiles is capturing the relative activity of a gene across different tissues, we propose a normalization strategy to highlight this property and allow easy visualization. Although genes located in close proximity often have similar expression profiles, their absolute expression levels can differ by several orders of magnitude (Ravarani *et al.* 2016, Faure *et al.* 2017, Morgan and Marioni 2018, Sigalova *et al.* 2020, Einarsson *et al.* 2022). To address this, we focused on estimating the relative shape (distribution) of the expression profiles rather than their absolute values. First, we applied a log transformation (West 2022) to compress the range between low-expressing tissues (e.g. 0 TPM) and high-expressing ones (e.g. 267 405 TPM) to make profiles more comparable. Second, we performed *z*-score standardization (Andrade 2021) to center each profile at a mean of zero with a standard deviation of one, minimizing the impact of large-scale variations and reduce the influence of extreme values. Finally, to ensure all values were positive, we normalized the standardized data to a 0–1 range.

### 2.4 Implementing greedy algorithm and branch and bound algorithm to find set of reference genes

We obtained the standard implementations of the greedy algorithm and branch and bound algorithm to solve the traditional set cover problem from this GitHub repository: https://github.com/AndreaRubbi/Set-Cover-problem-solution-Python. The standard implementation assigns a cost (e.g. number of elements in the set) to each set and finds the optimal solution by minimizing this cost. To adapt the code for our vicinity set cover problem, we modified the source code to treat the sets as having uniform cost (i.e. 1).

## 3 Results and discussion

### 3.1 Estimating gene expression profile based on closer vicinity shows less error

**Figure 1.**
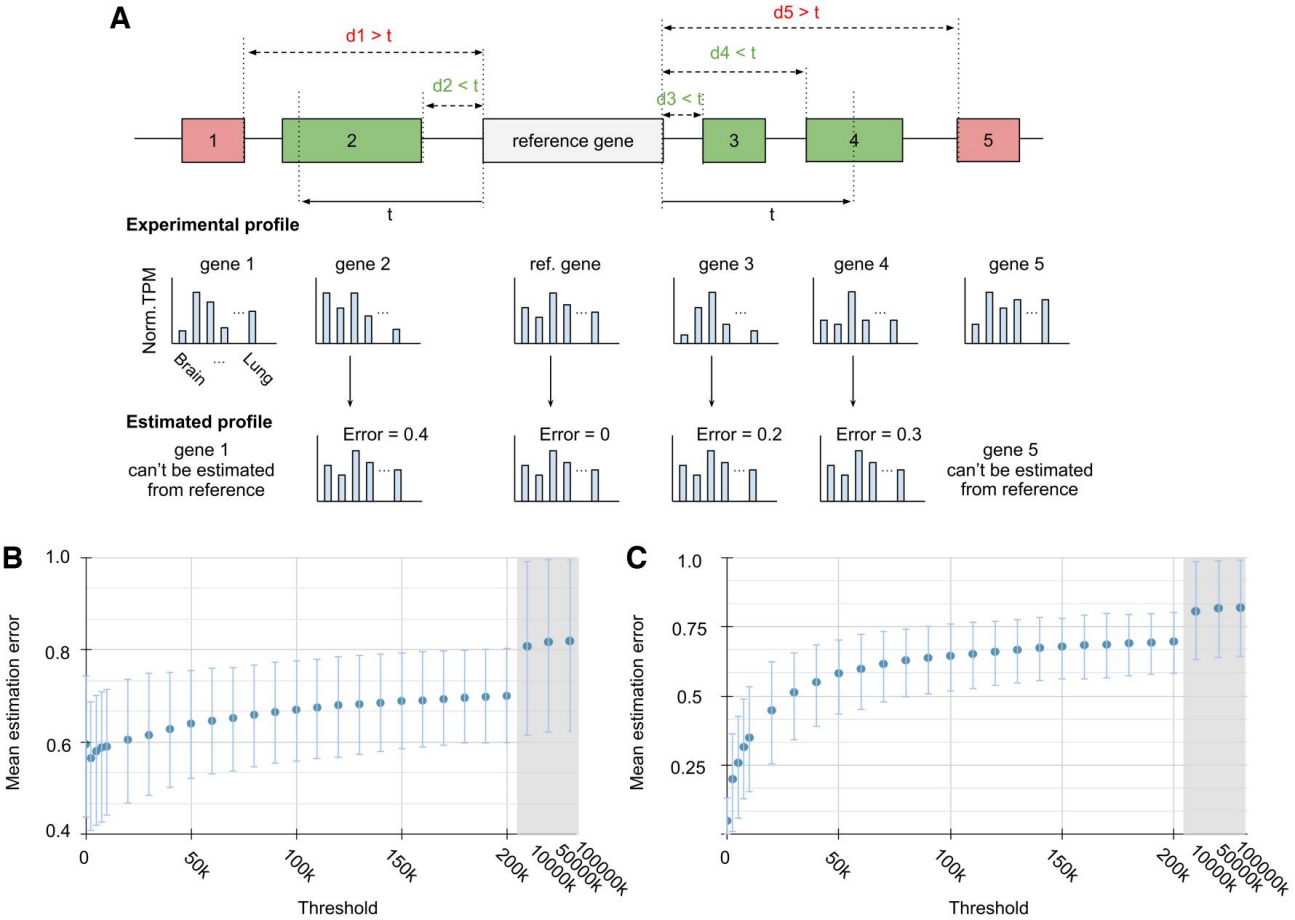
Estimating gene expression profile using a reference gene. (A) When threshold = *t*, the vicinity of a reference gene (white gene) includes gene 2, 3, and 4 (green genes). The expression profiles of the green genes can be estimated to be identical to the reference gene since their respective distances (d2, d3, and d4) are less than t. The expression profiles of genes 1 and 5 (red genes) cannot be estimated from the reference because their distances (d1 and d5) are >t. (B) Graph of average estimation error versus vicinity threshold of only estimated profiles. Note: a threshold of 0 corresponds to cases where genes overlap or are nested. (C) Graph of average estimation error versus vicinity threshold of all profiles.

The expression profile of a gene can be estimated using the expression profile of a reference gene if it is within a vicinity defined by a proximity threshold (Fig. 1). The distance between a gene and its reference gene is calculated as the difference between the transcriptional start sites (TSS) if the genes are on the same orientation, or between the TSS and the transcription end site (TES) if they are on different orientations. Specifically, for a threshold of t, the vicinity of a reference gene is defined as the genomic region spanning t base pairs (bps) upstream of its TSS and t bps downstream of its TES. Consequently, the vicinity of a reference gene includes all genes within a total distance of 2t bps, excluding the length of the reference gene itself. Additionally, if a gene is partially within the vicinity, it also belongs to that vicinity (e.g. gene 2 and 4 in Fig. 1a). When a gene is within the vicinity of a reference gene, its expression profile is estimated to be identical to the reference gene. As the threshold increases, more distal genes are included in the vicinity and thus, the estimation accuracy is expected to decrease. This is because closely located genes have more similar expression profiles than distant ones (Dong *et al.* 2024). To quantify expression similarity between genes, the commonly used metric is the Pearson correlation coefficient (PCC) (Huminiecki and Wolfe 2004, Liao and Zhang 2006, Miller and Bishop 2021). For this study, the PCC is calculated between the experimental and estimated expression profiles of a gene, and the estimation error is defined as 1-PCC. Since PCC ranges from −1 to 1, this estimation error ranges from 0 to 2, where 0 is identical correlated and 2 is inversely correlated. Typically, 1-PCC < 0.6 is considered moderately correlated (Evans 1996). Overall, smaller estimation errors indicate greater similarity in expression profiles, which is expected for genes in closer proximity, while larger errors are anticipated for genes further apart.

There is a clear tradeoff between average estimation error and vicinity threshold: smaller thresholds yield lower estimation error but require more experimentally determined profiles (Fig. 1b and c). To quantify this relationship, we calculated the average estimation error across a range of thresholds spanning small to large distances (e.g. 2500, 5000, 7500, 10k, 20k, …, 200k bps). For each threshold, the average estimation error was calculated genome-wide, where n represents the number of genes and m denotes the number of genes within a given vicinity:

average estimation error = 1n∑in(1m∑jm 1-PCC(genei,generef))       (2)

As expected, the average estimation error for genes that require estimation (i.e. non-reference genes) increases with higher thresholds and eventually plateaus at 0.8 (Fig. 1b) (i.e. j ≠ reference gene). However, to reflect the overall accuracy of genome-wide expression profiling, we must also account for experimentally determined genes. While their estimation error is effectively zero, they require direct measurement, which is costly and what we aim to minimize (Fig. 1a). At a threshold of zero, nearly all expression profiles are from reference genes, resulting in minimal estimation error (Fig. 1c). As the threshold increases, more profiles are estimated rather than experimentally determined, leading to higher estimation errors but requiring fewer experiments. At very large thresholds (e.g. 10 000 kbps, 50 000 kbps, 100 000 kbps), the average estimation error approaches a value slightly above 0.85, which is consistent with previous studies (Dong *et al.* 2024). To statistically compare average estimation errors across thresholds, we performed a one-way ANOVA followed by Tukey’s HSD post-hoc test (Fig. 1c). Significant differences were observed between distant thresholds (e.g. t = 10k bps versus t = 20k bps, *P* = 1.0; 10k bps versus 50k bps, *P* = .5368; 0 bps versus 70 kbps, *P* = .0005). Additionally, we further validated this trend using the MESA mouse expression dataset, which contains 126 tissue-specific expression profiles. As with the human genome, estimation error increased with larger thresholds and plateaued around 0.93 at extreme distances (see Fig. 3, available as supplementary data at *Bioinformatics Advances* online).

Gene co-expression similarity is influenced by more than just genomic proximity. Even at the smallest threshold of 2500 bps, estimation error remains nonzero, indicating that additional biological factors impact similarity. For example, certain transcriptional strands and directionality influence whether genes on the same strand share regulatory elements or face divergent regulation due to antisense transcription, potentially disrupting similarity (Ling *et al.* 2020). Regulatory elements such as enhancers that activate distant genes or insulators that block interactions can either enhance or interrupt co-expression (Fujioka *et al.* 2021). Chromatin domains like topologically associating domains promote co-regulation within spatial regions but isolate genes across boundaries, affecting similarity beyond linear distance (Dixon *et al.* 2012). Tissue-specific regulatory effects driven by unique transcription factors cause expression similarity to vary across tissues (Sonawane *et al.* 2017). Integrating these mechanisms could translate to adjusting thresholds or constraining specific reference genes in our estimation (e.g. more open chromatin around a gene might result in choosing a larger threshold). However, a carefully designed strategy is essential to effectively incorporate these factors.

### 3.2 Finding a set of reference genes through the set cover problem

**Figure 2.**
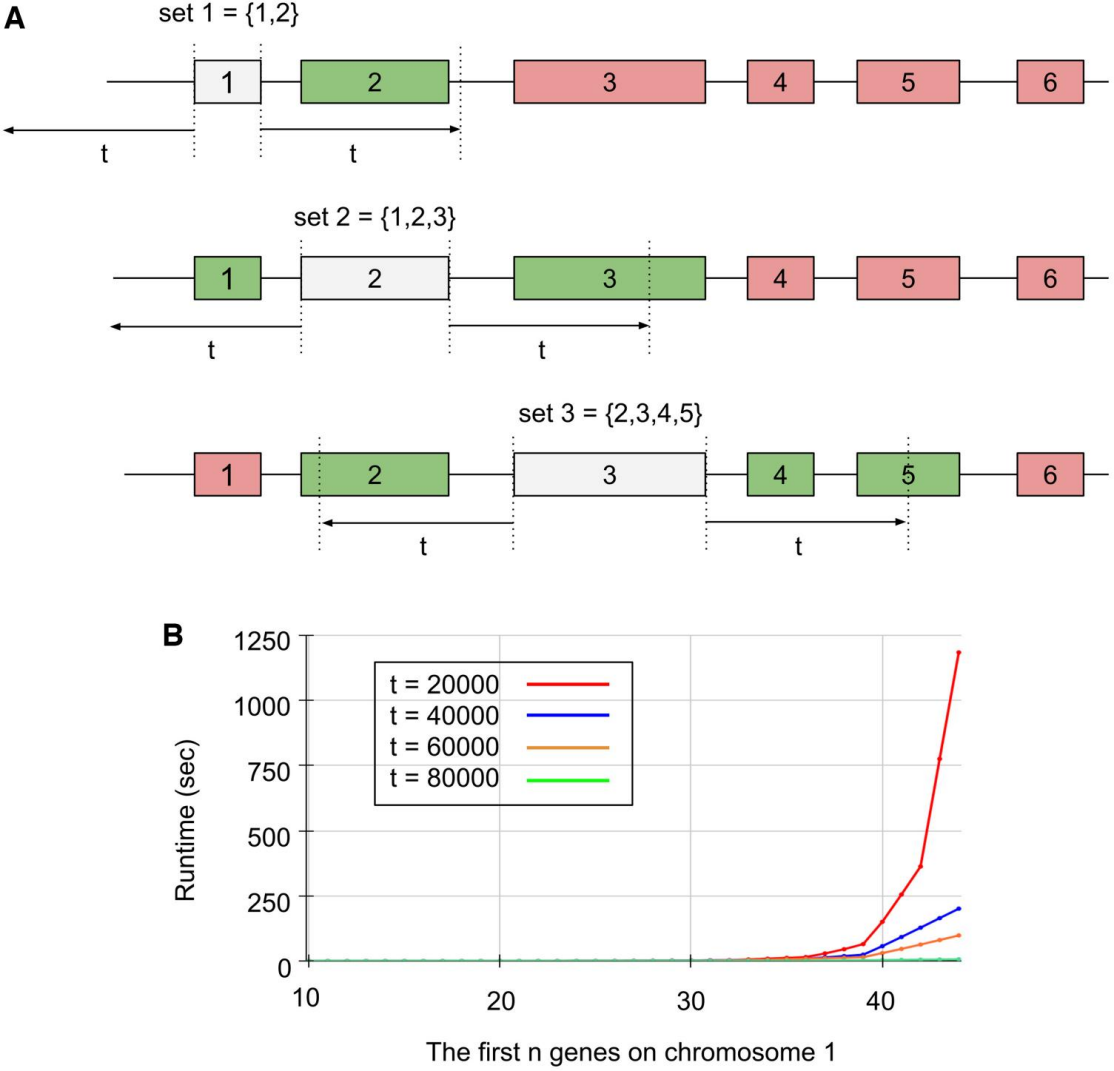
Mapping vicinity to gene sets and graph of runtime of the branch and bound algorithm to the set cover problem. (A) When threshold = *t*, gene 1’s set contains itself and gene 2; gene 2’s set contains itself and gene 1 and gene 3; gene 3’s set contains itself and gene 2, gene 4, and gene 5. (B) Graph of the runtime taken by the branch and bound algorithm to find the optimal solution using the first n gene sets on chromosome 1 at various thresholds (e.g. 20k, 40k, 60k, 80k bps).

By mapping the vicinity of a threshold into a gene set, the solution to the set cover problem can find a set of reference genes that allows the estimation of all profiles in the genome, but the branch and bound algorithm is too slow, and the greedy algorithm is non-optimal (Fig. 2). The vicinity of a reference gene can be mapped to a gene set where the genes within its vicinity become the elements. The union of all such gene sets collectively covers the entire genome. Given the profile of a reference gene, we can estimate the profile for all genes within its gene set by assuming identity to the reference (Fig. 2a). As there are overlapping elements between gene sets, it is possible that a larger gene set covers the same number of elements as multiple smaller gene sets. Therefore, we can obtain the optimal number of gene sets that can cover the entire genome through an exact solution to the set cover problem. We implemented and compared both the greedy algorithm and the branch and bound algorithm to find a set of reference genes that allows the estimation of all profiles in the genome. As expected with an increasing number of genes, the greedy algorithm has polynomial runtime while the branch and bound algorithm have exponential runtime (Fig. 2b). To evaluate the scalability, we applied the branch and bound algorithm to the genes on chromosome 1. While it completed for a small number of genes (e.g. fewer than 20 genes), it took approximately 8 h to process just the first 60 of 1880 genes on chromosome 1, showing the exponential runtime limitations of exact methods on large datasets (Fig. 2b). Despite the speed advantage of the greedy algorithm, its approximate solutions can include more gene sets than necessary. To illustrate a case where the greedy algorithm fails to achieve an optimal solution compared to branch and bound, we tested both algorithms at t = 20k bps using just the first 34 gene sets on chromosome 1. The branch and bound algorithm identified the optimal solution with 11 gene sets, whereas the greedy algorithm required 12 gene sets (see Fig. 1, available as supplementary data at *Bioinformatics Advances* online). This result shows that although the greedy algorithm is fast, it already does not give the optimal solution using just a small part of the dataset.

### 3.3 Finding a set of reference genes through the vicinity set cover problem by a dynamic programming algorithm

**Figure 3.**
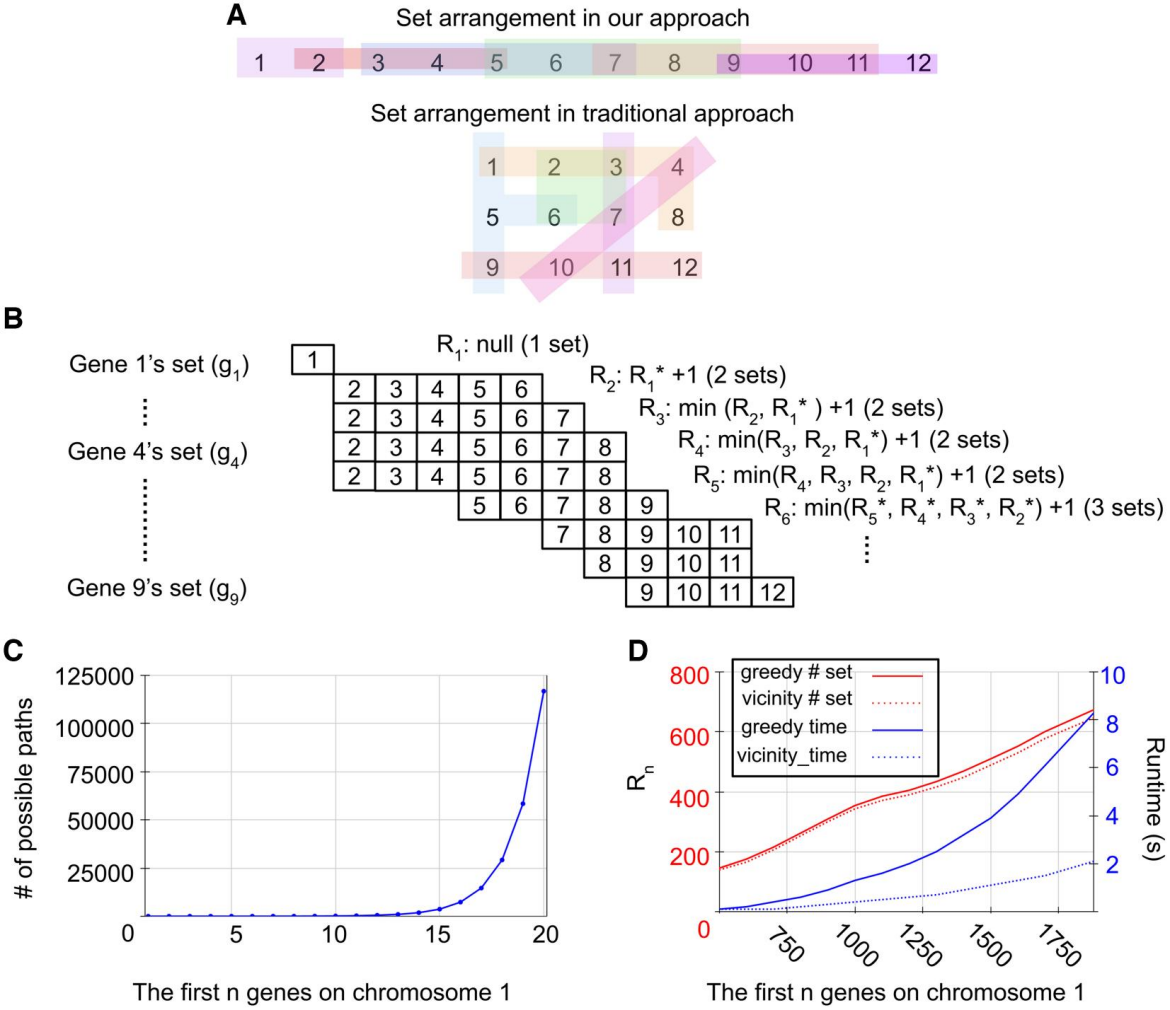
Solving the vicinity set cover problem by dynamic programming. (A) The elements are consecutively ordered in our algorithm compared to unordered in the traditional set cover problem. (B) Example of the algorithm’s flow during the calculation of *S*. (* indicates the optimal path) (C) Graph showing the number of possible paths with a minimizing criterion. (D) Graph comparing *R_n_* and runtime between the greedy set cover algorithm and our vicinity set cover algorithm. The representative threshold was 60k bps.

Since the gene sets of a vicinity consist of consecutively ordered genes, we can find the optimal solution using a dynamic programming algorithm that solves a variant of the set cover problem which we call the vicinity set cover problem (Fig. 3a). In the traditional set cover problem, there are no restrictions on the ordering of elements within a set (i.e. any element of the universe can be grouped into a set). In our vicinity set cover problem, the locations of genes are ordered in the genome such that a gene set can only include consecutively ordered genes because of their genomic locations. For example, a valid gene set in the vicinity set cover problem must contain consecutively ordered elements such as {5,6,7,8,9} but not {4,7,10} because it skips genes 5, 6, 8, and 9 (Fig. 3a). Furthermore, in the traditional set cover problem, there is no ordering of the sets in the solution. In our vicinity set cover problem, there is an ordering of sets in the solution which we call a path of sets. That is, to arrive at the optimal path to a particular gene set, $g_{i}$, we track the possible paths from preceding gene sets and choose the one with the fewest gene sets as it would mean fewer experiments (Fig. 3b). A possible preceding gene set cannot have a gap in the genes because the genes in gaps would have no profile estimated by a reference gene. For example, {2,3,4,5,6}, {2,3,4,5,6,7}, {2,3,4,5,6,7,8} are gene sets that can precede {5,6,7,8,9} but the {1} gene sets cannot precede since it has a gap of missing genes {2,3,4}. Note that gene sets can be identical when genes have identical neighbors (e.g. $g_{4}$ and $g_{5}$ both cover genes 2 to 8).

To define variables in our vicinity set cover problem, consider a genome with n protein coding genes, indexed from the first gene (i.e. 1) to the last gene (i.e. n) based on their genomic location (Table 1). For each gene i, we define a gene set $g_{i}$ that includes all genes located within a specified vicinity determined by a threshold t. The ordered collection of all such gene sets, starting with g_1_ and ending with $g_{n}$, is denoted as $G=[g_{1},\ldots ,g_{n}]$. For a given gene set $g_{i}$, the optimal path set, $S_{i}$, is determined. An optimal path set to $g_{i}$ includes the minimum number of gene sets from $g_{1}$ to $g_{i}$, which covers all genes up to the ith gene of the genome. Since there can be multiple optimal path sets, $S_{i}$ is a set of optimal path sets. As the total number of gene sets in each optimal path set of $S_{i}$ is the same value, that value is represented by $R_{i}$.

**Table 1.** Defining the variables and their corresponding descriptions.

Notation	Description
n	The number of protein-coding genes in a genome
i	Current gene index i=1…n
$g_{i}$	A gene set of the ith gene’s neighborhood defined by threshold t bps
G	An ordered collection of all gene sets in the genome by position, where $g_{1}$ is the first gene, and $g_{n}$ is the last gene. $G=[g_{1},g_{2},\ldots ,g_{n}]$
$P_{i}$	All possible path sets from $g_{1}$ to $g_{i}$
$S_{i}$	The path sets that include the minimum number of gene sets from $g_{1}$ to $g_{i}$, which covers all genes up to the ith gene of the genome
$R_{i}$	The number of gene sets in the i-th path(s)

To solve our vicinity set cover problem, we propose Algorithm 1. Consider the example where we have $G=[g_{1},g_{2},\ldots ,g_{9}]$ (Fig. 3b). Our algorithm begins by assigning $S_{1}$ with one optimal path that includes just $\left[[g_{1}]\right]$. Then, it incrementally computes $S_{2}$,…,$S_{i}$,…,$S_{n}$. To arrive at $S_{i}$, there can be multiple possible path sets. Without a criterion to minimize the number of the possible path sets, the number of path sets increases exponentially (Fig. 3c, Fig. 2, available as supplementary data at *Bioinformatics Advances* online). Since the minimal number of experiments is desired, the optimal path sets have the minimal number of gene sets, and this criterion forms the recurrence computation in our dynamic programming algorithm. After $S_{n}$ is computed, all the optimal path sets are determined and each path set has the minimal number of gene sets to cover the entire genome. In our example, we first assign $S_{1}=[[g_{1}]]$ and compute $R_{1}=1$. At $g_{2}$, $S_{2}=[S_{1}\;\cup \;[g_{2}]]$ (element wise union) and $R_{2}=2$. At $g_{3}$, $S_{3}$ can be either from $S_{1}$ or $S_{2}$. Since the R from $S_{1}$ (i.e. $R_{1}=1$) is shorter than $S_{2}$ (i.e. $R_{2}=2$), then $S_{3}=[S_{1}\;\cup \;[g_{3}]]$ and $R_{3}=2$. At $g_{6},\;S_{6}$ can be either from $S_{2}$, $S_{3}$, $S_{4}$, $S_{5}$. Since the R from $S_{2}$, $S_{3}$, $S_{4}$, $S_{5}$ are the same, then $S_{6}=[S_{2}\;\cup \;[g_{6}],\;S_{3}\;\cup \;[g_{6}],\;S_{4}\;\cup \;[g_{6}],\;S_{5}\;\cup \;[g_{6}]]$ and $R_{6}=3$. In this way, we break down a larger problem into smaller subproblems where we minimize the number of gene sets in the path sets at each iteration, ensuring scalability by discarding non-optimal paths. The order complexity of the algorithm is O($n^{2}$) because the number elements in $P_{\min}$ is at worst case n (i.e. case with large thresholds of vicinity). However, since thresholds are typically much shorter than the length of the genome, the algorithm’s runtime is closer to O(n).

Algorithm 1: Find minimum number of sets that covers the genome.

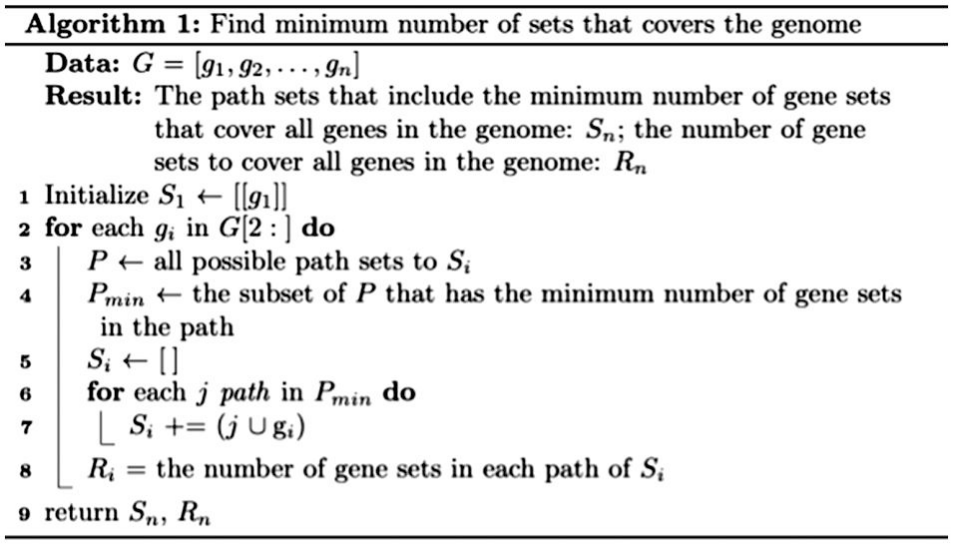

To verify the correctness of our algorithm, we first compared its results with those of the branch and bound algorithm with various gene counts (i.e. the first n genes in chromosome 1) (Fig. 3d). At every gene count tractable by the branch and bound algorithm, both produced the same $R_{n}$ in the solution. Since the branch and bound algorithm becomes intractable quickly, we then compare our algorithm with the greedy algorithm on various gene counts to evaluate its solution and speed (Fig. 3d). As expected, the greedy algorithm consistently has more gene sets in the solution than our algorithm, while our algorithm also outperforms the greedy algorithm in speed (Fig. 3, available as supplementary data at *Bioinformatics Advances* online). These phenomena were also observed in other thresholds (e.g. 20k, 40k, 80k bps).

### 3.4 Finding the most optimal path set

**Figure 4.**
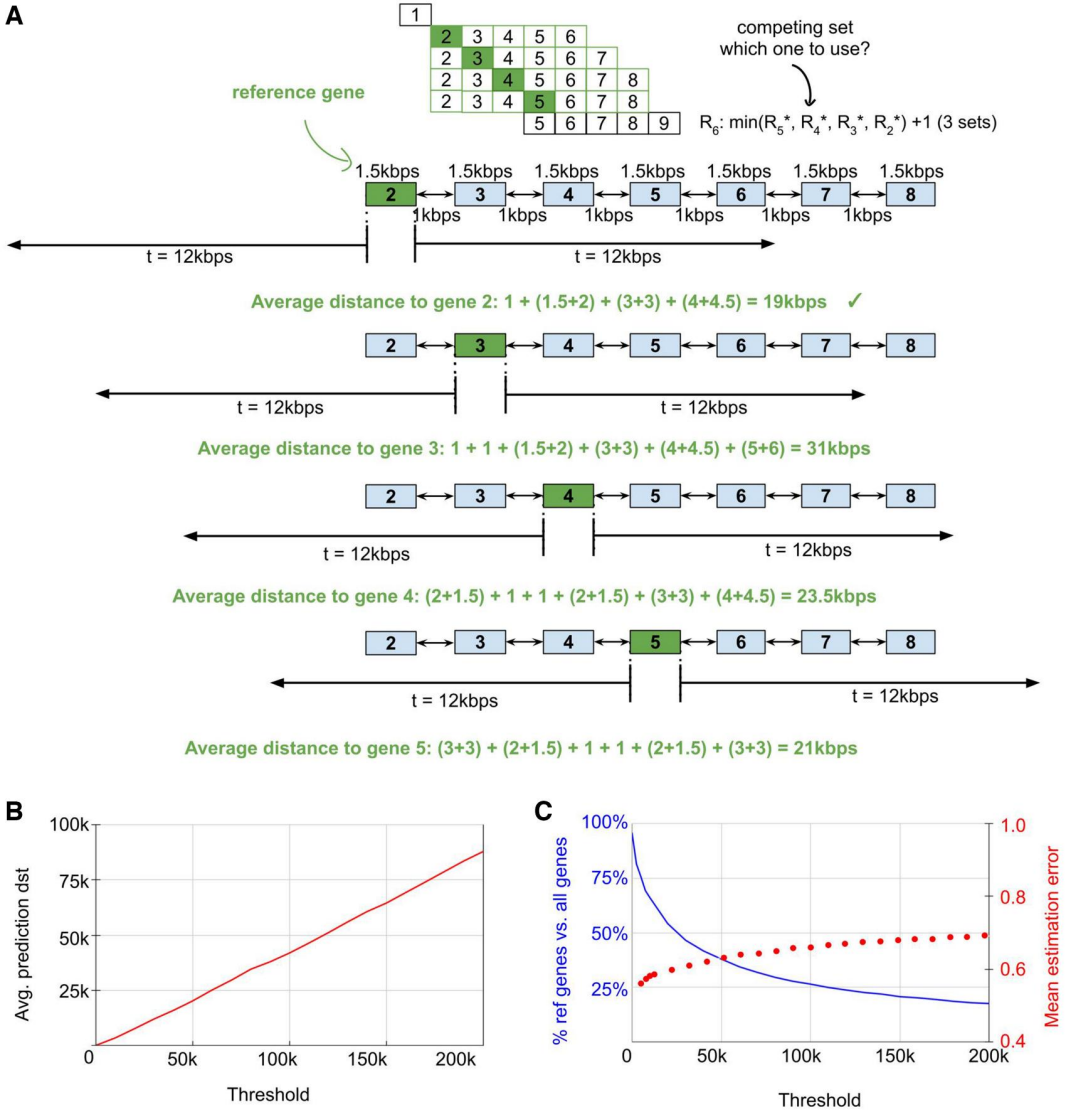
Reducing the size of the optimal path sets by calculating the average distance to the reference gene. (A) Graph showing a case where multiple optimal paths occur. Each gene is 1.5 kbps long and separated by 1 kbps. The threshold used is 12 kbps. (B) Graph showing as the thresholds increase, the average estimation distance within each gene set grows proportionally. (C) Graph showing with increasing threshold, the proportion of reference genes relative to the total number of genes gradually decreases. The mean estimation error shows the same trend as in Fig. 1b.

Since there can be multiple optimal paths (i.e. different paths that have the same number of gene sets) in $S_{i}$, the single path that is most optimal is defined as the path set with the smallest average distance between vicinity genes to reference genes because shorter distances lead to higher estimation accuracy (Fig. 4a). Using the gene locations, we can calculate the average estimation distance in $g_{i}$ that contains m genes in the vicinity using the following equation:

average estimation distance = 1m∑j=1m({|TESj-TSSref| if j<ref|TSSref-TESj| if j>ref)       (3)

Now, only the most optimal path set is kept in $S_{i}$. For example, there are four optimal paths to $S_{6}$ but the most optimal path is from $S_{2}$, as it has the smallest average distance (i.e. 19k bps) (Fig. 4a). When making estimations, we observed that the average estimation distance increases proportionally to the selected threshold, which is equal to roughly half of the threshold value (Fig. 4b). As the threshold increases, fewer reference genes are required to estimate the remaining genes but there is a decrease in estimation accuracy (Figs 4c and 1c). For example, at larger thresholds (e.g. t = 200k bps), only 330 reference genes (Fig. 4c) are required to be experimentally determined on chromosome 1 (1880 genes) but the estimation accuracy is low (i.e. mean estimation error = 0.70 ± 0.10) (Fig. 1c). At smaller thresholds (e.g. *t* = 50k bps), more reference genes (i.e. 713) (Fig. 4c) are required on chromosome 1 but the estimation accuracy is higher (i.e. 0.64 ± 0.12) (Fig. 1c). As extremely small thresholds (e.g. 0 bps), almost all genes are needed as reference genes (i.e. 1801) (Fig. 4c) and only the overlapped or nested genes are estimated at a very high accuracy (i.e. 0.05 ± 0.08) (Fig. 1c). When this extremely small threshold is applied to all protein-coding genes (18 078 genes) in the human genome, this avoids the need for experimental work on 777 genes. For thresholds (e.g. 2500, 5000, 7500, 10k, 20k, …, 200k bps), the reference gene profiles were used for estimation of vicinity gene profiles and as expected, the mean estimation error is similar to Fig. 1b, suggesting that the selection of optimal reference genes robustly retains the fact that close vicinity genes have similar profiles (Fig. 4c).

## 4 Conclusion

The similarity in genotype tissue expression profile among genes in close vicinity suggests that genome-wide expression profiles could be estimated using a small set of reference genes, reducing the need for extensive experiments. By defining a vicinity using a proximity threshold around a reference gene, the expression profile of a gene in the vicinity can be estimated using the reference gene. To identify these reference genes, we mapped gene vicinities into sets and applied the traditional set cover algorithms to obtain the optimal number of gene sets that can cover the entire genome. However, traditional set cover algorithms are either too slow or produce non-optimal results for large datasets. Given that genes within a vicinity are consecutively ordered, we develop a dynamic programming algorithm to solve this vicinity set cover problem within a tractable runtime. Since there could be multiple optimal solutions with the same number of gene sets, the most optimal solution was determined as the one with the smallest average distance between vicinity genes to reference genes because shorter distances enhance estimation accuracy. This could be particularly valuable for reducing extensive experiments in organisms where there is no genotype tissue expression or another human dataset with an expanded set of tissues (e.g. non-coding RNAs). Likewise, this algorithm can be adapted to data derived from single-cell datasets. Beyond genomics, our algorithm has broader applications in optimization problems involving consecutive ordered sets, such as optimizing phased array antenna placement for maximum coverage and minimal interference. This algorithm not only reduces experimental workload but also offers a tractable solution for similar challenges in other fields. Future studies could refine the selection of thresholds by leveraging factors such as transcriptional strand and directionality, regulatory elements (e.g. enhancers and insulators), chromatin domains like TADs, and tissue-specific regulatory effects.

## Supplementary Material

vbaf163_Supplementary_Data

## Data Availability

The data underlying this article are available in “vicinity_set_cover” repository at: https://github.com/sensationTI/vicinity_set_cover.

## References

[vbaf163-B1] Agathos S , Papapetrou E. On the set cover problem for broadcasting in wireless ad hoc networks. Commun Lett IEEE 2013;17:2192–5.

[vbaf163-B2] Andrade C. Z scores, standard scores, and composite test scores explained. Indian J Psychol Med 2021;43:555–7. 35210687 10.1177/02537176211046525 PMC8826187

[vbaf163-B3] Boschetti M , Maniezzo V. A set covering based matheuristic for a real-world city logistics problem. Int Trans Oper Res 2015;22:169–95.

[vbaf163-B4] Caprara A , Toth P, Fischetti M et al Algorithms for the set covering problem. Ann Oper Res 2000;98:353–71.

[vbaf163-B5] Carniel A , Mestria M. Optimization methods to solve the set covering problems. Contrib LAS Cienc Soc 2023;16:17784–807.

[vbaf163-B6] Chvatal V. A greedy heuristic for the set-covering problem. Math OR 1979;4:233–5.

[vbaf163-B7] Dey SS , Dubey Y, Molinaro M et al Lower bounds on the size of general branch-and-bound trees. Math Program 2023;198:539–59.

[vbaf163-B8] Dixon JR , Selvaraj S, Yue F et al Topological domains in mammalian genomes identified by analysis of chromatin interactions. Nature 2012;485:376–80. 22495300 10.1038/nature11082 PMC3356448

[vbaf163-B9] Dong J , Brown S, Truong K et al Nearby and non-nested genes in the human genome have more similar genotype tissue expression. PLoS One 2024;19:e0307360. 39292702 10.1371/journal.pone.0307360 PMC11410254

[vbaf163-B10] Einarsson H , Salvatore M, Vaagensø C et al Promoter sequence and architecture determine expression variability and confer robustness to genetic variants. Elife 2022;11:e80943. 36377861 10.7554/eLife.80943 PMC9844987

[vbaf163-B11] Evans JD. Straightforward Statistics for the Behavioral Sciences. Belmont, CA, US: Thomson Brooks/Cole Publishing Co, 1996.

[vbaf163-B12] Faure AJ , Schmiedel JM, Lehner B et al Systematic analysis of the determinants of gene expression noise in embryonic stem cells. Cell Syst 2017;5:471–84.e4. 29102610 10.1016/j.cels.2017.10.003

[vbaf163-B13] Fujioka M , Nezdyur A, Jaynes JB et al An insulator blocks access to enhancers by an illegitimate promoter, preventing repression by transcriptional interference. PLoS Genet 2021;17:e1009536. 33901190 10.1371/journal.pgen.1009536 PMC8102011

[vbaf163-B14] GTEx Consortium The Genotype-Tissue expression (GTEx) project. Nat Genet 2013;45:580–5. 23715323 10.1038/ng.2653 PMC4010069

[vbaf163-B15] Heger A , Holm L. Picasso: generating a covering set of protein family profiles. Bioinformatics 2001;17:272–9. 11294792 10.1093/bioinformatics/17.3.272

[vbaf163-B16] Hou Z , Jiang P, Swanson SA et al A cost-effective RNA sequencing protocol for large-scale gene expression studies. Sci Rep 2015;5:9570. 25831155 10.1038/srep09570 PMC4381617

[vbaf163-B17] Huminiecki L , Wolfe KH. Divergence of spatial gene expression profiles following species-specific gene duplications in human and mouse. Genome Res 2004;14:1870–9. 15466287 10.1101/gr.2705204 PMC524410

[vbaf163-B18] Klemm SL , Shipony Z, Greenleaf WJ et al Chromatin accessibility and the regulatory epigenome. Nat Rev Genet 2019;20:207–20. 30675018 10.1038/s41576-018-0089-8

[vbaf163-B19] Krishnaswamy R et al Online and dynamic algorithms for set cover. In: STOC 2017 Proceedings of the 49th Annual ACM SIGACT Symposium on Theory of Computing. ACM. 2017, 537–50.

[vbaf163-B20] Li Y , Liu Y, Juedes D et al Set cover-based methods for motif selection. Bioinformatics 2020;36:1044–51. 31665223 10.1093/bioinformatics/btz697 PMC7703758

[vbaf163-B21] Liao B-Y , Zhang J. Low rates of expression profile divergence in highly expressed genes and tissue-specific genes during mammalian evolution. Mol Biol Evol 2006;23:1119–28. 16520335 10.1093/molbev/msj119

[vbaf163-B22] Ling JP , Wilks C, Charles R et al ASCOT identifies key regulators of neuronal subtype-specific splicing. Nat Commun 2020;11:137. 31919425 10.1038/s41467-019-14020-5 PMC6952364

[vbaf163-B23] McCombie WR , McPherson JD, Mardis ER et al Next-generation sequencing technologies. Cold Spring Harb Perspect Med 2019;9:a036798. 30478097 10.1101/cshperspect.a036798 PMC6824406

[vbaf163-B24] Miller HE , Bishop AJR. Correlation AnalyzeR: functional predictions from gene co-expression correlations. BMC Bioinformatics 2021;22:206. 33879054 10.1186/s12859-021-04130-7 PMC8056587

[vbaf163-B25] Morgan MD , Marioni JC. CpG island composition differences are a source of gene expression noise indicative of promoter responsiveness. Genome Biol 2018;19:81. 29945659 10.1186/s13059-018-1461-x PMC6020341

[vbaf163-B26] Ravarani CNJ , Chalancon G, Breker M et al Affinity and competition for TBP are molecular determinants of gene expression noise. Nat Commun 2016;7:10417. 26832815 10.1038/ncomms10417 PMC4740812

[vbaf163-B27] Savage SR , Shi Z, Liao Y et al Graph algorithms for condensing and consolidating gene set analysis results. Mol Cell Proteomics 2019;18:S141–S152. 31142576 10.1074/mcp.TIR118.001263 PMC6692773

[vbaf163-B28] Sigalova OM , Shaeiri A, Forneris M et al Predictive features of gene expression variation reveal mechanistic link with differential expression. Mol Syst Biol 2020;16:e9539. 32767663 10.15252/msb.20209539 PMC7411568

[vbaf163-B29] Sonawane AR , Platig J, Fagny M et al Understanding tissue-specific gene regulation. Cell Rep 2017;21:1077–88. 29069589 10.1016/j.celrep.2017.10.001 PMC5828531

[vbaf163-B30] Stoney RA , Schwartz J-M, Robertson DL et al Using set theory to reduce redundancy in pathway sets. BMC Bioinformatics 2018;19:386. 30340461 10.1186/s12859-018-2355-3 PMC6194563

[vbaf163-B31] West RM. Best practice in statistics: the use of log transformation. Ann Clin Biochem 2022;59:162–5. 34666549 10.1177/00045632211050531 PMC9036143

