# Supplementary material for: Optimal solution to the set cover problem with a vicinity constraint for estimating genotype tissue expression profiles
Source: Bioinform Adv. 2025 Jul 4;5(1):vbaf163. doi: 10.1093/bioadv/vbaf163 (PMC12313015; doi:10.1093/bioadv/vbaf163)
Supplement: vbaf163_Supplementary_Data [file vbaf163_supplementary_data.docx]

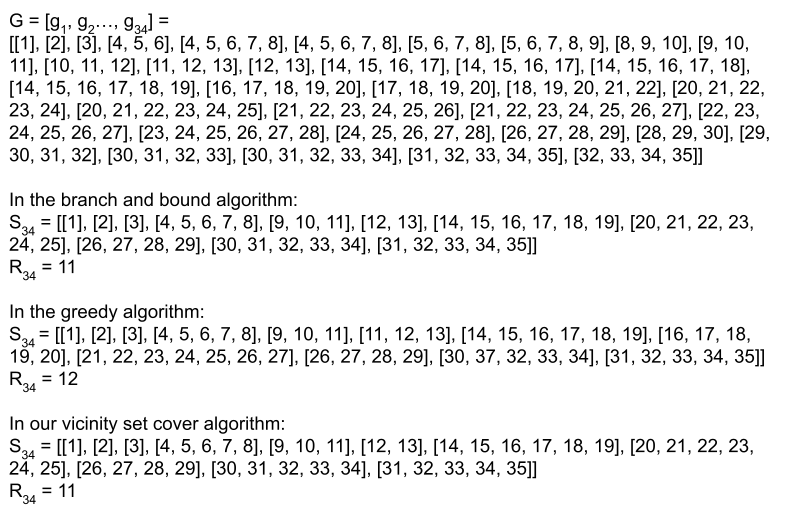


**Supplementary Figure S1.** Example comparison of solutions from the greedy algorithm, the branch-and-bound method, and our vicinity set cover algorithm, using the first 35 genes on chromosome 1 with a vicinity threshold of 20 kbps.


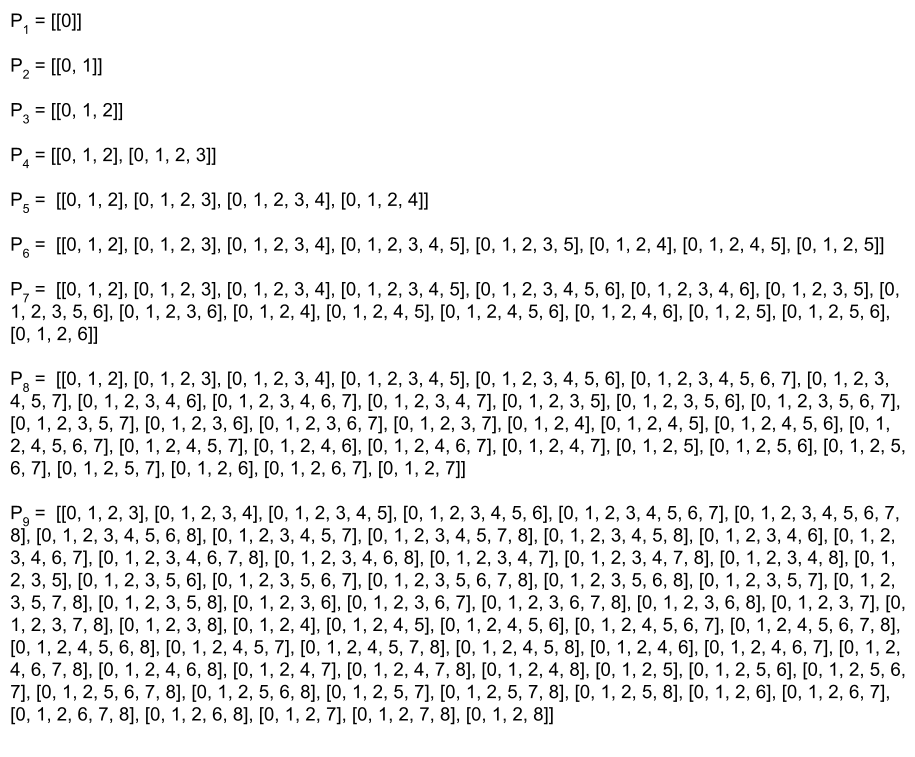


**Supplementary Figure S2.** An example showing the number of paths grows exponentially if not optimized.


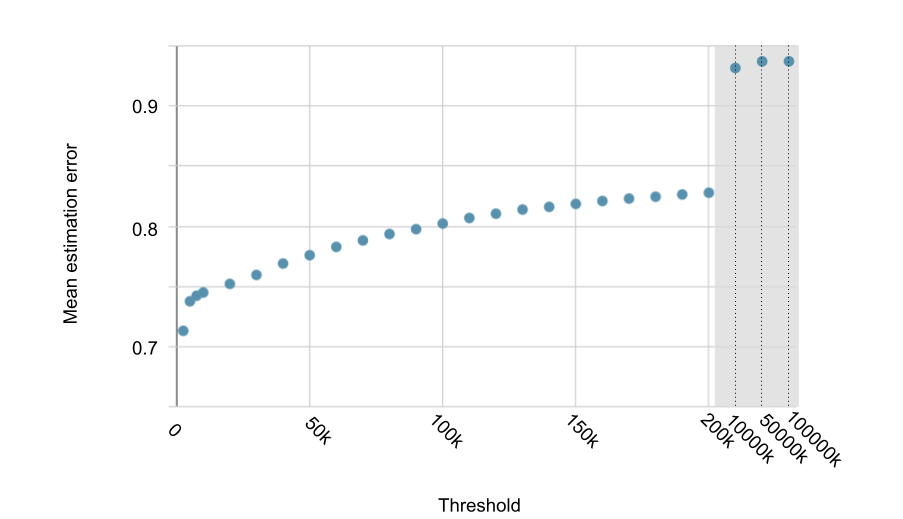


**Supplementary Figure S3.** Estimating gene expression profile in the mouse genome. Graph of average estimation error vs vicinity threshold of only estimated profiles. Note: a threshold of 0 corresponds to cases where genes overlap or are nested.
